# The Impact of Recreational Diving to a Depth of 40 m on Selected Intracellular DAMPs

**DOI:** 10.3390/ijms26073061

**Published:** 2025-03-27

**Authors:** Anna Nowakowska, Małgorzata Marchelek-Myśliwiec, Marta Skórka-Majewicz, Wojciech Żwierełło, Konrad Grzeszczak, Izabela Gutowska

**Affiliations:** 1 Institute of Biology, University of Szczecin, Wąska 13, 71-415 Szczecin, Poland; 2 Clinical Department of Nephrology, Transplantology & Internal Medicine, Pomeranian Medical University in Szczecin, 70-111 Szczecin, Poland; malgorzata.marchelek.mysliwiec@pum.edu.pl; 3 Department of Medical Chemistry, Pomeranian Medical University in Szczecin, 70-111 Szczecin, Poland; marta.skorka.majewicz@pum.edu.pl (M.S.-M.); wojciech.zwierello@pum.edu.pl (W.Ż.); 4 Department of Laboratory Diagnostics, Pomeranian Medical University, 70-111 Szczecin, Poland; konrad.grzeszczak@pum.edu.pl

**Keywords:** damage-associated, molecular patterns, antioxidant markers, recreational diving

## Abstract

Increasingly popular, recreational diving is a physical activity that takes place under extreme environmental conditions, which include hyperoxia, hyperbaria and exposure to cold water. The effects of these factors on the human body induce increased levels of reactive oxygen and nitrogen species in divers’ bodies, which may modulate damage-associated molecular pattern (DAMPs), their receptors and the antioxidant response. This study involved 21 divers who descended to a depth of 40 metres. Determinations of selected intracellular DAMPs (high-mobility group box protein 1,HMGB1, S100 calcium-binding proteins A9 and A8, S100A8 and S100A9, heat shock protein family A member 1A, HSPA1A (Hsp70), heat shock protein family B, (small) member 1, HSPB1(Hsp27), thioredoxin, TXN), their receptors (Toll-like receptor 4, TLR4 and receptors for advanced glycation end products, RAGE), nuclear factor-κB (NF-κB) and antioxidant defence markers were performed before, after and 1 h after the dive. A significant transient reduction in HMGB1 expression was observed immediately after the dive at both the mRNA and protein levels. We noted an increase in S100A9 expression, which occurred 1 h post-dive compared to the post-dive time point, and a post-dive decrease in TLR4 expression only at the mRNA level. Diving also influenced the expression of genes encoding key enzymes associated with glutathione synthesis, (glutamate-cysteine ligase, catalytic subunit, GCLC and glutathione synthetase, GSS), and reduced plasma glutathione levels. However, no significant changes were observed in the expression of NF-κB, nitric oxide synthase 2 (NOS2) or circulating DAMP receptors (TLR4 and RAGE). The findings suggest an adaptive response to diving-induced oxidative stress, which appears to be a protective mechanism against an excessive inflammatory response. To our knowledge, this is the first study to analyse the role of intracellular DAMPs in recreational divers.

## 1. Introduction

Diving has become a popular recreational activity in Poland. It is recognized as a form of moderate physical activity conducted in extreme environmental conditions. High pressure, increased inspired partial O_2_ pressure (pO_2_) and low water temperature during diving affect organ function and induce transient metabolic changes [1]. Recreational divers have a depth limit of 40 m. This is the maximum depth at which it is generally possible to ascend without requiring decompression. They use compressed air or Nitrox (a mixture of oxygen and nitrogen) as breathing gases, with the oxygen content not exceeding 40% [2]. Breathing compressed air is only feasible up to a depth of approximately 50 m. This limitation is due to the risk of oxygen toxicity at greater depths [3].

Oxygen is responsible for forming reactive oxygen species (ROS), which are responsible for generating oxidative stress. Under normal physiological conditions, approximately 1% to 2% of inhaled O_2_ is directly converted to ROS. However, this rate increases in response to the hyperoxia that occurs during diving [4]. A state of oxidative stress damages lipids, proteins and the DNA of cells and can also affect endothelial function. It is known that excessive accumulation of ROS can lead to cell damage and death [5]. On the other hand, ROS also function as important signalling molecules, essential for cell viability, playing various regulatory roles [6]. Endothelial dysfunction and oxidative stress have been extensively studied in diving [4,7,8]. As a result of hyperbaric and hyperoxic conditions during diving, ROS production increases. These molecules function as signalling agents, inducing cellular adaptations that improve oxidative stress tolerance. For example, the antioxidant enzyme thioredoxin reductase has increased activity in breath-hold divers [9,10]. In SCUBA (Self-Contained Breathing Apparatus) divers, the hyperoxia-induced rise in ROS activates the expression of various antioxidant enzymes [11]. The glutathione system also plays a role in ROS balance and is affected in SCUBA and saturation divers [12,13,14] as well as during hyperbaric oxygen therapy (HBO) [15].

Diving also induces nitrosative damage, known as nitrosative stress. A rapid ascent at the end of a dive can cause barotrauma in the lungs and sinuses. As ambient pressure drops, nitrogen absorbed during the dive is released from body fluids, forming gas bubbles in the blood and tissues [16,17]. Nitric oxide (NO) is a free radical produced in vivo by three isoforms of NO synthase. The three isoforms are neuronal NOS (type I), inducible NOS (type II) and endothelial NOS (type III). Since oxygen is a necessary substrate for NO synthesis, hyperoxia increases the activity of all three NOS isoforms [4]. ROS reacting with NO produces ONOO^−^(peroxynitrite), which impairs NO-mediated vasodilation and endothelial function [18]. Furthermore, peroxynitrite exacerbates oxidative stress by increasing xanthine oxidase activity and impairing antioxidant defenses [19,20].

Reactive nitrogen species (RNS), primarily nitric oxide and peroxynitrite, play key roles in regulating inflammation in the human body [21]. These molecules regulate HMGB1 (high-mobility group box protein 1) metabolism and its receptors, among other physiological processes [22,23,24,25,26]. HMGB1 is classified as a damage-associated molecular pattern (DAMP). DAMPs are endogenous molecules released from the extracellular matrix or dying cells [27]. Intracellular DAMPs are molecules released during the breakdown of necrotic and apoptotic cells, including calcium-binding proteins S100, HMGB1, thioredoxin (TXN), heat shock proteins (HSPs) and uric acid. In contrast, extracellular DAMPs consist of components of the extracellular matrix, such as glycoproteins, proteoglycans and glycosaminoglycans [27,28,29]. The biological activity of DAMPs is mediated by pattern recognition receptors (PRRs), including Toll-like receptors (TLRs) and receptors for advanced glycation end products (RAGE) [30]. Both hyperoxia and physical exertion induce intracellular oxidative stress, which can increase TLR expression in various cell types, leading to the release of inflammatory cytokines [31,32]. TLR4, a member of the TLR family, can be recruited by peroxynitrite [25,26,33]. The interaction of DAMPs with pattern recognition receptors initiates inflammatory cascades, activating multiple transcription factors, including nuclear factor-κB (NF-κB), a key regulator of the inflammatory response [34]. Moreover, the NF-κB pathway also plays a role in HMGB1 release. Suppressing the canonical NF-κB pathway reduces HMGB1 secretion in activated immune cells [35,36,37].

HMGB1 is a non-histone nuclear protein that functions both intracellularly and extracellularly. Many studies indicate that ROS play a crucial role in the active secretion and passive release of HMGB1 in various cell types [5]. In addition to inducing active HMGB1 secretion, ROS can promote its passive release during different forms of cell death [38]. HMGB1 activity has been observed in skeletal muscle and the central nervous system. In vitro studies have shown an increase in HMGB1 levels in muscle fibres and infiltrating leukocytes during skeletal muscle regeneration, coinciding with the antioxidant response [39]. HMGB1 is a recognized biomarker of post-exercise inflammation [40]. Study outcomes indicate that HMGB1 can undergo oxidation in the cytoplasm by ROS [41]. Studies suggest that after oxidation, HMGB1 is converted into a pro-inflammatory agent that induces an inflammatory response via TLR4 [42].

In addition to HMGB1, TLR4 can serve as a receptor for other DAMPs, such as heat shock proteins and S100 calcium-binding proteins [43]. RAGE is another receptor for DAMPs that recognizes HMGB1 and S100 proteins [44,45]. Analysis of the transcriptome of experienced divers showed that prolonged dives led to persistent changes in cellular signalling pathways, including HSP60/HSP70 activation via TLR4 and pathways mediated by NF-κB [46]. In this study, we investigated the expression of selected intracellular DAMPs, their receptors and NF-κB in relation to markers of antioxidant defence in recreational divers who performed dives to a depth of 40 m.

## 2. Results

**Figure 1:**
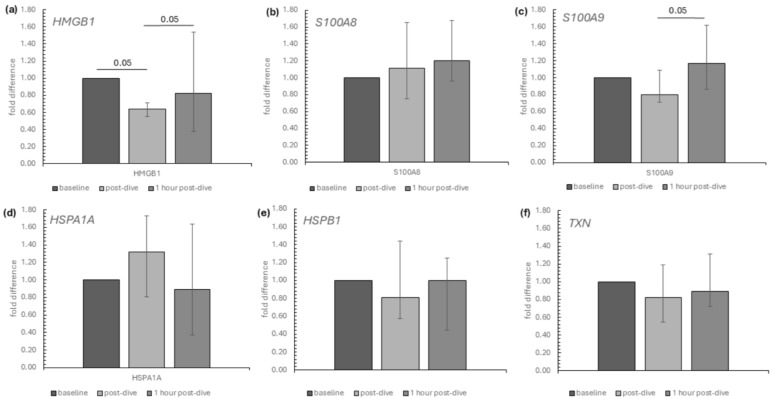
The influence of diving to a depth of 40 m on the relative mRNA expression of (**a**) HMGB1, (**b**) S100A8, (**c**) S100A9, (**d**) HSPA1A, (**e**) HSPB1 and (**f**) TXN in the group of recreational divers (medians and interquartile range). The data are presented as fold differences. Significance levels of differences observed between analysed time points (baseline vs. post-dive vs. 1 h post-dive) were assessed using Friedman’s analysis of variance followed by post-hoc Dunn’s test. HMGB1—high-mobility group box protein 1, S100A8—S100 calcium-binding protein A8, S100A9—S100 calcium-binding protein A9, TXN—thioredoxin, HSPB1—heat shock protein family B, (small) member 1, HSPA1A—heat shock protein family A (Hsp70) member 1A.

The influence of diving to a depth of 40 m on the relative mRNA expression of the studied genes encoding selected intracellular DAMPs in the group of recreational divers is presented in Figure 1. Among intracellular DAMPs, dives triggered a significant transient reduction in the mRNA relative expression of HMGB1. On the other hand, S100A9 mRNA expression significantly rose between the post-dive and 1 h post-dive points (Figure 1a,c). A Friedman analysis of baseline, immediate post-dive and 1 h post-dive results showed no statistically significant changes in the mRNA relative expression in the rest of the analysed intracellular DAMPs (S100A8, HSPA1A, HSPB1, TXN) (Figure 1b,d–f).

**Figure 2:**
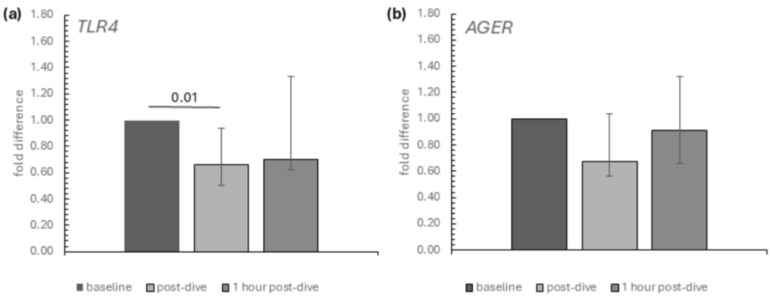
The influence of diving to a depth of 40 m on the relative mRNA expression of (**a**) TLR4 and (**b**) AGER in the group of recreational divers (medians and interquartile range). The data are presented as fold differences. Significance levels of differences observed between analysed time points (baseline vs. post-dive vs. 1 h post-dive) were assessed using Friedman’s analysis of variance followed by post-hoc Dunn’s test. TLR4—Toll-like receptor 4, AGER—advanced glycosylation end-product-specific receptor.

Within the DAMP potential receptors selected for this study, we noted that only the mRNA relative expression of TLR4 was affected by dives. It was significantly decreased immediately after the dive, while no statistically significant difference was observed between baseline and 1 h post-dive values. The decline in AGER’s relative expression (gene encoding receptor RAGE) was not statistically significant (Figure 2a,b).

**Figure 3:**
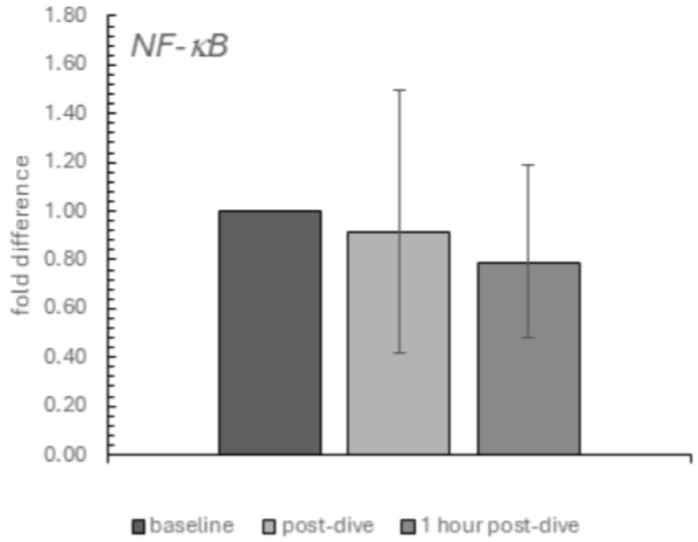
The influence of diving to a depth of 40 m on the relative mRNA expression of the NF-κB in the group of recreational divers (medians and interquartile range). The data are presented as fold differences. Significance levels of differences observed between analysed time points (baseline vs. post-dive vs. 1 h post-dive) were assessed using Friedman’s analysis of variance followed by post-hoc Dunn’s test. NF-κB—nuclear factor-κB.

Similarly, the decrease in the relative mRNA expression of NFκB was also insignificant (Figure 3).

**Figure 4:**
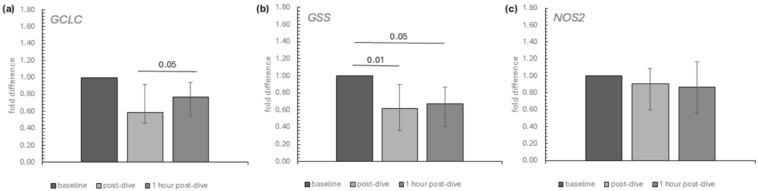
The influence of diving to a depth of 40 m on the relative mRNA expression of (**a**) GCLC, (**b**) GSS and (**c**) NOS2 in the group of recreational divers (medians and interquartile range). The data are presented as fold differences. Significance levels of differences observed between analysed time points (baseline vs. post-dive vs. 1 h post-dive) were assessed using Friedman’s analysis of variance followed by post-hoc Dunn’s test. GCLC—glutamate-cysteine ligase, catalytic subunit, GSS—glutathione synthetase, NOS2—nitric oxide synthase 2.

Within selected genes encoding proteins linked to antioxidant status, we observed a transient decrease and a significant increase in GCLC (glutamate-cysteine ligase) mRNA expression at the 1 h post-dive time point compared to immediately post-dive (Figure 4a). The mRNA relative expression of GSS (glutathione synthetase) was diminished at the analysed time points after dives in comparison to baseline (Figure 4b). Both genes encode enzymes necessary for glutathione synthesis (Figure 4a,b). On the other hand, dives did not significantly affect the relative expression of NOS2 (Figure 4c).

**Figure 5:**
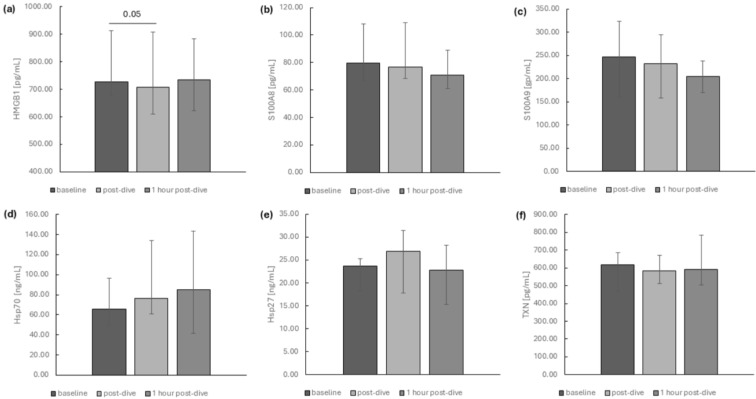
The influence of diving to a depth of 40 m on (**a**) HMGB1, (**b**) S100A8, (**c**) S100A9, (**d**) Hsp70, (**e**) Hsp27 and (**f**) TXN protein levels in the group of recreational divers (medians and interquartile range). Significance levels of differences observed between analysed time points (baseline vs. post-dive vs. 1 h post-dive) were assessed using Friedman’s analysis of variance followed by post-hoc Dunn’s test. HMGB1—high-mobility group box protein 1, S100A8—S100 calcium-binding protein A8, S100A9—S100 calcium-binding protein A9, Hsp27 (HSPB1)—heat shock protein family B, (small) member 1, Hsp70 (HSPA1A)—heat shock protein family A member 1A, TXN—thioredoxin.

The impact of dives to a depth of 40 m on the selected intracellular DAMP protein levels in recreational divers’ plasma is shown in Figure 5. Except for the transient decline of HMGB1 protein level immediately after dives (Figure 5a), we did not note any significant differences in studied protein concentrations after performing Friedman analysis of variance (S100A8, S100A9, TXN, HSPA1A, HSPB1) (Figure 5b–f).

**Figure 6:**
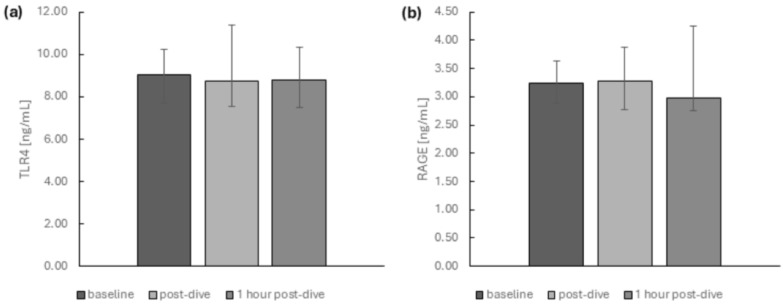
The influence of diving to a depth of 40 m on the protein levels of (**a**) TLR4 and (**b**) RAGE in the group of recreational divers (medians and interquartile range). Significance levels of differences observed between analysed time points (baseline vs. post-dive vs. 1 h post-dive) were assessed using Friedman’s analysis of variance followed by post-hoc Dunn’s test. TLR4—Toll-like receptor 4, RAGE—receptor for advanced glycation end products.

Selected DAMP receptors—soluble forms of TLR4 and RAGE—did not significantly differ between time points after diving (Figure 6).

**Figure 7:**
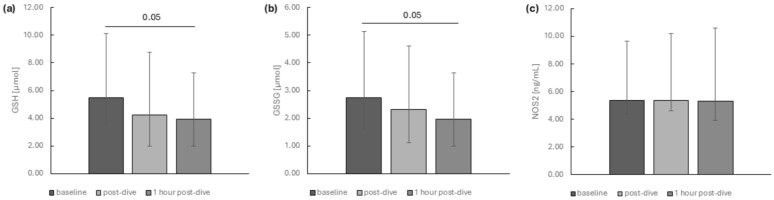
The influence of diving to a depth of 40 m on the levels of the (**a**) GSH, (**b**) GSSG and (**c**) NOS2 in the group of recreational divers (medians and interquartile range). Significance levels of differences observed between analysed time points (baseline vs. post-dive vs. 1 h post-dive) were assessed using Friedman’s analysis of variance followed by post-hoc Dunn’s test. GSH—reduced glutathione, GSSG—glutathione disulfide (oxidised glutathione), NOS2—nitric oxide synthase 2.

We noted that the concentrations of the reduced and oxidised forms of glutathione in plasma successfully declined at the analysed time points (Figure 7a,b).

**Figure 8:**
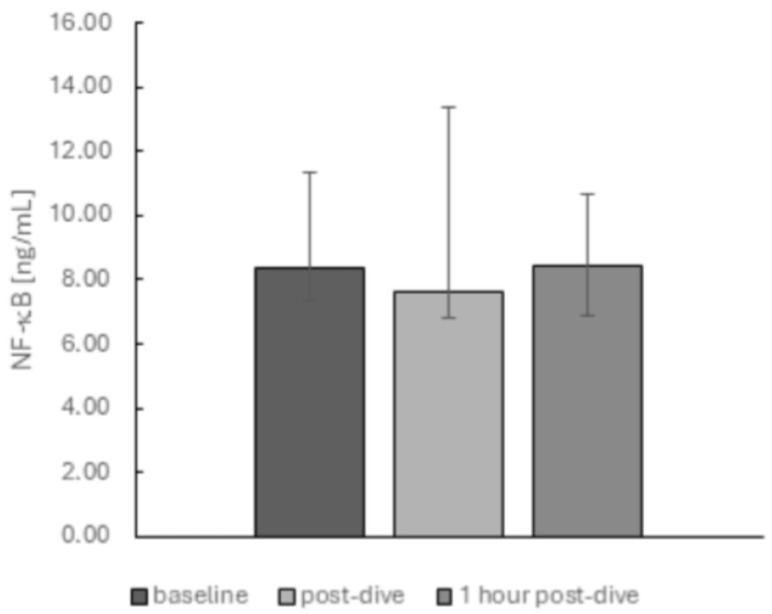
The influence of diving to a depth of 40 m on the NFκB protein level in the group of recreational divers (medians and interquartile range). Significance levels of differences observed between analysed time points (baseline vs. post-dive vs. 1 h post-dive) were assessed using Friedman’s analysis of variance followed by post-hoc Dunn’s test. NF-κB—nuclear factor-κB.

We did not observe any changes in NOS2 (Figure 7c) and NFκB (Figure 8) protein levels.

## 3. Discussion

Characterized by hyperoxia, hyperbaria and moderate exertion, diving triggers an inflammatory response [10,11]. This immune response, known as ‘sterile inflammation’, is a low-grade inflammatory reaction triggered by environmental, mechanical or stress factors [47]. Although inflammation is essential for tissue repair, unresolved chronic inflammation can contribute to inflammatory diseases [28,47,48].

We demonstrated the transient decline of damage-associated molecular pattern, HMGB1, at both the gene and protein levels in recreational SCUBA divers. Among other studied DAMPs, we noted only a significant increase in S100A9 mRNA expression at the 1 h post-dive time point compared to immediately post-dive. Within the DAMP receptors selected for this study, we noted that only the mRNA relative expression of TLR4 significantly decreased immediately after the dive. The dives to a depth of 40 m did not influence the soluble forms of TLR4 and RAGE receptor levels in the recreation divers’ blood. We did not observe any significant changes in NFκB expression, neither on the gene nor protein level.

Reactive oxygen and nitrogen species through cellular damage contribute to HMGB1 leakage. Depending on the concentration of ROS and RNS and the duration of exposure, HMGB1 proteoforms with different biological activities are formed [49]. HMGB1 can exist in three redox states that significantly affect its half-life and function: the fully reduced form, the disulphide form and the oxidised form with sulfonated cysteines. The first two are active, while the oxidised form is inactive and predominates when inflammation is suppressed. The fully reduced form activates inflammatory pathways through RAGE, while the disulphide form is the most stable and acts through TLR4 [50]. Typically, increased oxidative stress leads to the oxidation and inactivation of HMGB1. Also, HMGB1 itself, by binding to its receptor, may contribute to increased oxidative stress and its inactivation. For example, activation of RAGE receptors by HMGB1 in eosinophils accumulating at necrotic sites stimulates eosinophil peroxidase to generate ROS that inhibit the function of this protein [51]. On the other hand, results from some studies indicate that ROS-induced modification of HMGB1 in specific situations does not necessarily lead to a loss of its biological activity. For example, studies on ageing cell lines showed that oxidation of the cysteine residues of HMGB1 improved its ability to bind to TLR4 and increased the release of inflammatory cytokines [52].

Hyperoxia typically increases the expression of HMGB1 [24,53,54], as well as HMGB1-binding receptors, both TLR4 [25,26,33,55] and RAGE [55,56]. The consequence of this condition is TLR4- and NF-κB-dependent activation of cellular pathways leading to an increase in inflammatory cytokine secretion [57,58]. Hyperoxia-induced activation of TLR4 can, for example, lead to brain tissue damage [59] or pathological myocardial remodelling [60]. NF-κB is also involved in hyperoxia-dependent activation of the RAGE receptor [54,56,58]. Results from studies in knockout mice with genes for TLR4 and RAGE showed that these receptors play a key role in HMGB1-induced inflammatory responses by regulating each other. On the one hand, RAGE is involved in the translocation of TLR4 to the cell membrane without affecting its transcription; on the other hand, TLR4 affects both the membrane transport of RAGE and its transcription. HMGB1 generates cytokine production by increasing the expression of both receptors and leading to activation of MAPK (mitogen-activated protein kinases) pathways [61].

However, in our study, where the time from exposure to diving-related hyperoxia to the assays was relatively short, we did not observe significant changes in RAGE and NF-κB expression at the mRNA or protein level. However, we observed a transient decrease in mRNA for HMGB1 and a decrease in mRNA for TLR4 immediately after the dive. A study on mouse endothelial cells isolated from the lung showed that the hyperoxia-associated increase in RAGE expression at the protein level was accompanied by a decrease in mRNA levels for RAGE four hours after exposure compared with cells cultured in normoxia [56]. A similar situation was observed in our study concerning mRNA expression for TLR4. However, in contrast to the cell line studies, we did not observe changes in protein levels for either TLR4 or RAGE. This is most likely related to the limitation of our study to the determination of soluble forms in blood due to its preliminary nature. For both receptors, soluble forms are formed by the proteolytic action of metalloproteinases. TLR4 is cut by ADAM10/17 (a disintegrin and metalloproteinase domain-containing protein 10/17) [62], whereas RAGE is formed by the action of MMP-9 (matrix metalloproteinase-9) and ADAM10 [63]. In addition, approximately 25% of circulating RAGE is its variant formed by an alternative splicing pathway, endogenous secretory RAGE (esRAGE). The production of esRAGE is induced by the interaction of membrane-anchored RAGE with its ligands (also with DAMPs, e.g., HMGB1 and S100 proteins), which leads to the activation of signalling pathways associated with the activation of the transcription factor NF-κB [63]. Soluble forms are thought to be structurally identical to their membrane-bound counterparts but do not participate in the pathways initiated by these receptors [62]. Soluble forms act as decoy receptors for both TLR4 [64] and RAGE [65]. This involves preemptively binding ligands of these receptors (including DAMPs) and blocking their interaction with receptors located in cell membranes, resulting in inhibition of their activity and reduced expression of inflammatory genes [55,62,65,66]. In metabolic diseases, the circulating form of RAGE is regarded as a specific biomarker of ligand–RAGE pathway overactivity. The lower the circulating level of soluble RAGE, the higher the activity concerning ligand binding to the membrane-bound receptor and the worse the prognosis [67,68]. Thus, the lack of change in soluble RAGE levels in our study seems to demonstrate the lack of effect of diving to a depth of 40 m on the interactions between membrane-bound RAGE receptors and their ligands in such a limited study time. In contrast, circulating soluble TLR4 is formed by proteolytic cleavage of membrane TLR4, secretion from the intracellular pool and alternative splicing [69,70]. Given the many factors potentially contributing to the formation of plasma levels of soluble TLR4, no correlation between membrane-localised TLR4 and soluble TLR4 is noted [69]. In our study, we did not observe significant fluctuations in soluble TLR4 levels; however, mRNA relative expression for TLR4 significantly decreased after diving.

Most underwater conditions such as hyperoxia and hyperbaria can be simulated in the hyperbaric chamber. Studies in animals and humans following acute spinal cord injury indicate that the use of hyperbaric oxygen therapy (HBO) is associated with a significant reduction in the expression of TLR4, NF-κB and HMGB1 at both mRNA and protein levels [71,72,73] as well as RAGE [74]. A similar effect was produced by the application of HBO in rabbits with osteoarthritis, where a reduction in HMGB1 expression in chondrocytes was noted. This effect was explained by the effect of HBO on increasing miR-107 expression, which in turn inhibited HMGB1 and its dependent receptors RAGE and TLR4 as well as NF-κB activation [54]. The mechanism for the downregulation of HMGB1 expression by hyperbaric oxygen therapy is also explained by its induced increase in sirtuin1 (SIRT1) expression. In an in vitro study in a mouse model of ischaemic stroke, HBO was observed to inhibit HMGB1 via SIRT1-dependent deacetylation [75]. In contrast, a study of a 30 min recreational dive to a depth of 30 m after a winter non-diving period showed that not SIRT1 but SIRT3 expression is stimulated in response to oxidative stress. A substantial, significant increase in mRNA expression for SIRT3 was reported 6 h after the dive, while SIRT1 expression decreased [76].

Of the intracellular DAMPs we studied, we observed a transient decrease and a significant increase in S100A9 mRNA expression at the 1 h post-dive time point compared to immediately post-dive. DAMP-belonging S100 proteins act through TLR4 and RAGE receptors to promote the inflammatory response [43,45,77]. By acting through RAGE, they may also contribute to increased ROS production [78]. S100 proteins are markers of many inflammatory diseases [79]. It appears that hyperbaric oxygen therapy exerts an inhibitory effect on these proteins and may alleviate their course. For example, in the treatment of femoral head osteoarthritis, it reduces serum levels of S100A9 protein, thus contributing to improved tissue vascularisation [80,81], while in Crohn’s disease, it has a down-regulating effect on S100A8/S100A9 heterotetramers (calprotectin) [82]. A post-exercise increase in S100 proteins, associated with tissue repair processes, is also noted. For example, plasma levels of the S100A8/A9 complex increase in many types of exercise, such as high-intensity interval training, treadmill running, cycling and dynamic exercise [83]. S100 proteins may also provide important information on tissue damage associated with decompression illness (DCI). Divers who completed four consecutive non-decompression dives to a depth of 18 metres recorded a significant increase in S100b post-dive, which was most likely related to skeletal muscle rather than central nervous system damage [84]. A transient increase in serum S100B levels was also observed in apnoeic divers, and this increase was comparable to that seen in patients after ischaemic stroke [85]. In our study, the expression of S100A9 and S100A8 proteins, although not statistically significant, seems to have a decreasing trend. Longer follow-up of our divers would perhaps yield significant results.

Also belonging to the intracellular DAMPs, the heat shock proteins Hsp70 (HSPA1A) and Hsp27 (HSPB1), which we studied, did not show significant changes in expression either at the mRNA or protein level. In experiments on hyperoxia-treated mice, heat shock proteins, particularly Hsp70, have a protective role against lung endothelial cells. There, Hsp70 is an important ligand for TLR4 and the adaptor protein Trif, whose activation contributed to increased survival in these animals [86]. In contrast, another protein of the heat shock protein family, Hsp70L1, is responsible for enhancing the response of Th1 helper lymphocytes precisely through TLR4 [87]. Intracellularly localised Hsp70 is known to have an immunosuppressive effect by inhibiting NF-κB. However, when it is located outside the cell, due to active or passive release, it can act as DAMPs through TLR2 and TLR4 and thus promote inflammation [88,89]. On the other hand, HSP induction may exert beneficial effects on endothelial cells. It has been observed, for example, that HSP activation is associated with reduced vesicle formation in decompression sickness [90]. In our study of recreational divers, we observed a slight upward trend for Hsp70, which would possibly have gained statistical significance if observation of recreational divers had been possible over a longer time interval. In opposition to our study, in professional divers performing simulated dives to depths of 30 m and 60 m in a hyperbaric chamber, a significant, substantial decrease in HSP70 levels was observed [91]. The opposite response may be due to the obvious better adaptation of the organisms of professional divers.

When examining recreational divers descending to a depth of 40 m, we found no significant changes in the expression of the last DAMP we analysed, thioredoxin. Hyperoxia leads to the induction of many antioxidant enzymes, including TXN [56]. During hyperbaric oxygen therapy, TXN as an antioxidant enzyme acts cytoprotectively by supporting other antioxidants in inactivating excess ROS and preventing redox imbalance. However, its priority function in HBO is related to its role in stabilising and stimulating the expression of hypoxia-inducible factors (HIFs), which are crucial for the formation of new blood vessels [92,93]. Thioredoxin reductase is closely associated with the maintenance of physiological TRX function. In repeatedly descending apnoeic divers whose bodies are subjected to alternating hypoxia and reoxygenation, an adaptive increase in the activity of this enzyme has been observed [9]. This is similar to the physiological responses of an organism subjected to exhaustive exercise, where post-exercise skeletal muscle recovery is associated with an increase in antioxidant enzymes such as superoxide dismutase (SOD) and TXN and an increase in free thiols [39]. Stimulatory effects on the levels of one of the more important thiols, total and reduced glutathione, may also be exerted by hyperbaric oxygen therapy [15]. On the other hand, a reduction in GSH levels associated with the generation of oxidative stress during exposure to HBO has been observed [94]. Studies on simulated diving and breath-hold diving also provide contrasting results. Simulated diving preceded by heat stress was reported to increase GSH levels by 62% [95], while a series of dives to a depth of 20 m for 20 min contributed to a reduction in thiol levels [18]. In our study, there was also a decrease in glutathione concentrations, both reduced and oxidised, and a decrease in the mRNA expression of one of the key enzymes to its synthesis—GSS. The mRNA expression of GCLC transiently decreased and significantly increased at the 1 h post-dive time point compared to immediately post-dive. Reduced levels of GSH and GCLC are characteristic of macrophages during the inflammatory response [96]. Oxidative stress associated with hyperoxia may also be the cause of the reduced GSH in our study. The hyperoxia state associated with diving increases the production of superoxide anion [97], which when converted to H_2_O_2_ reacts with NO to form peroxynitrite [98,99]. H_2_O_2_ is known to oxidise the thiol groups of cysteine [99], which may be the main reason for the reduced glutathione levels observed in our study. Our results indicate that the decrease in blood glutathione concentration may be related to the inhibitory effect of diving to a depth of 40 m on the expression of genes encoding enzymes involved in glutathione synthesis. In our study, the expression of the GSS gene was significantly lower immediately after the dive and one hour later.

The inducible nitric oxide synthase encoded by the NOS2 gene plays an important role in the body’s defence against oxidative stress. Heat shock proteins (Hsp70 and Hsp90), which activate the IKK-NF-κB signalling pathway [100], are required to initiate transcription of the NOS2 gene. Studies in sedentary rats have shown that NO produced by iNOS can support the defence of vascular endothelial cells against the attachment of bubble precursors to the vessel wall during simulated diving [101]. We did not observe significant changes in iNOS expression at mRNA and protein levels in the divers we studied descending to a depth of 40 m. Similarly, in a study by Sureda et al. where divers descended to a depth of 50 m, no changes in iNOS levels were recorded; however, indicative of the activity of this enzyme, nitrite levels in neutrophils increased after the dive and after a 3 h recovery period [10]. In another study, where divers simulated a descent to a depth of 60 m in a hyperbaric chamber, iNOS levels did not change [91] as in our study.

### Study Limitations and Perspectives

Our study focused exclusively on middle-aged divers due to the financial barriers associated with diving in Poland. The high cost of equipment makes the sport largely accessible to established professionals with stable incomes, which effectively limits access for younger people. Additionally, our research did not include female participants, as recreational diving in Poland remains male-dominated.

We measured intracellular DAMPs and other selected parameters on gene and protein levels at a small number of time points. Supposedly, downward or upward trends in protein concentrations and gene expression may reach statistical significance if the group is observed longer. Gathering a homogeneous and sufficiently large group of recreational year-round divers who would agree to stay at the dive base for an extended time and multiple blood draws is challenging. In the future, we intend to expand the study by including additional HMGB1-related proteins, refining our methodology and monitoring divers for longer periods.

## 4. Materials and Methods

### 4.1. Study Design and Participant Characteristics

Twenty-one recreational divers who dive regularly throughout the year were recruited for the study. Dives were carried out in Lake Ińsko (Ińsko Lake region, Western Pomerania, north-western Poland). The dives took place from June to July 2024 at a depth of 40 m and took a total of 50 min. The temperature of the surface layer of the lake ranged from 15 to 19 degrees Celsius, the water temperature up to 12 m was 19 degrees Celsius, the water temperature from 12 m—thermocline—was 8 degrees Celsius, the temperature near the bottom (about 40 m) varied from 5 to 6 degrees Celsius, and the air temperature varied from 25 to 30 degrees Celsius. Typically, diving operations took place between 10:00 and 12:00, with a descent to a maximum depth of 40 metres, followed by an immediate ascent to the surface without requiring a decompression stop. Diving equipment included open-circuit SCUBA diving equipment that used compressed air (21% O_2_) as a breathing gas, dive computers and dry suits.

Study participants underwent anamnestic examination to exclude acute or chronic diseases. The divers were non-smokers and were urged not to use drugs or food supplements before performing the dives. Before obtaining written consent, we informed all study participants of the risks and potential inconveniences associated with blood sampling. The study was conducted according to the guidelines of the Declaration of Helsinki and approved by the Bioethics Committee of the Pomeranian Medical University in Szczecin (no. KB-006/30/2022). The anthropometric characteristics of the participants in the study are presented in Table 1.

### 4.2. Blood Sampling

Blood samples intended for ELISA tests and RNA isolation were collected three times from the cubital vein: before diving (baseline), right after diving (post-dive) and 1 h following diving (1 h post-dive). Venous blood was gathered into 7.5 mL S-Monovette tubes containing an anticoagulant (EDTA K3, 1.6 mg EDTA/mL blood) and into 7.5 mL S-Monovette tubes with a clot activator (SAR-STEDT AG&Co., Nümbrecht, Germany). A portion of EDTA-anticoagulated blood for RNA isolation was promptly mixed with Lyse Blood buffer (EURx, Gdańsk, Poland), left at room temperature for 30 min, transported in a cooled container and subsequently stored at −80 °C, following the manufacturer’s instructions. Blood samples for ELISA testing were centrifuged at once at 3000 rpm for 10 min. The collected plasma and serum samples were transported in a cooled container and kept at −80 °C until they were processed.

### 4.3. ELISA

The plasma or serum levels of the proteins under study, including HMGB1, S100A8, S100A9, TXN, HSPA1A, HSPB1, NFκB, TLR4, RAGE and NOS2, were determined using the immunoenzymatic ELISA technique with commercial assay kits from ELK Biotechnology (Wuhan, China). The methodology followed the instructions provided by the manufacturer. Measurements of absorbance were taken using an Asys UVM340 microplate reader (Biochrom, Cambridge, UK) and MikroWin 2000 4.35 software in two replicates. The concentrations of the proteins HMGB1, S100A8, S100A9 and TXN were reported in pg/mL, whereas those of HSPA1A, HSPB1, NFκB, TLR4, RAGE and NOS2 were reported in ng/mL. GSH and GSSG levels were noted in µmol.

### 4.4. RNA Isolation and Reverse Transcription

Total RNA was isolated from peripheral blood leucocytes sourced from thawed blood samples utilising the Gene MATRIX Universal Blood RNA Purification Kit (EURx, Gdańsk, Poland) as per the manufacturer’s guidelines. This procedure included DNase I (EURx, Gdańsk, Poland) treatment to prevent genomic DNA contamination. Subsequently, the RNA samples’ concentration was measured using a BioTek Take3 Microvolume Plate and Synergy H1 Plate Reader (BioTek, Winooski, VT, USA) and Gen5 2.00 software. Then, 0.6 µg of total RNA from each sample was converted into cDNA using the Thermo Scientific RevertAid First Strand cDNA Synthesis Kit (Thermo Scientific, Waltham, MA, USA) in a 20 µL reaction volume, following the manufacturer’s instructions.

### 4.5. Real-Time qPCR Protocol

mRNA levels were analysed using qRT-PCR on a CFX96™ Touch Real-Time PCR Detection System (Bio-Rad, Hercules, CA, USA) and Bio-Rad CFX Manager 3.1 software with SG qPCR Master Mix (2×) from EURx, Gdańsk, Poland. Gene amplification was performed under the following conditions: an initial denaturation at 95 °C for 10 min, followed by 40 cycles at 94 °C for 15 s, at 60 °C for 30 s and at 72 °C for 30 s. The list of primers used for qPCR reactions is available in the supplementary materials (Table S1). Following qPCR, melting curve analysis was performed on all samples. Under these amplification conditions, a single PCR product was detected. The relative expression of the target gene, normalised to β2-microglobulin, was calculated using the 2^−∆∆Ct^ method to determine fold change and subjected to statistical analysis. Each sample was run in duplicate. 

### 4.6. Statistical Analysis

Statistical analyses were conducted utilising Statistica version 13 software (2017; TIBCO Software Inc., Palo Alto, CA, USA). A significance level of *p* < 0.05 was employed. Data were expressed as medians with interquartile ranges. Initially, we assessed data normality using the Shapiro–Wilk test. As the data were not normally distributed, we performed non-parametric statistical analysis. To evaluate differences across the three time points analysed (baseline vs. immediately post-dive vs. 1 h post-dive), we applied Friedman’s repeated-measure analysis of variance followed by Dunn’s post-hoc test.

## 5. Conclusions

For understandable reasons, professional diving has received more attention in diving research. However, due to the growing interest in this activity, recreational diving has begun to be recognised as a form of physical activity. It is therefore worth highlighting both the health risks and benefits associated with this activity. We observed a transient down-regulation of HMGB1 expression after diving at the mRNA and protein levels, as well as a reduction in TLR4 and an increase in S100A9 expression at the mRNA level only. These changes may indicate an adaptation of the divers against the oxidative stress accompanying diving, which was reflected in our study by a decrease in the levels of glutathione and the expression of the gene encoding one of the key enzymes responsible for its synthesis—GSS. The results of our study may contribute to a better understanding of how recreational diving affects the immune response in the context of oxidative stress, potentially offering insights into its long-term impact on health and adaptation. To our knowledge, this is the first study to investigate the role of intracellular DAMPs in the inflammatory response in recreational divers.

## Figures and Tables

**Table 1:** Anthropometric characteristics of the study participants (median and interquartile range).

	Recreational Diver Group N = 21
age [years]	50.0 (48.00−61.00)
height [cm]	1.80 (1.76−1.81)
weight [kg]	89.5 (85.0−92.5)
BMI [kg/m^2^]	27.8 (26.5−28.7)

N—number of participants, BMI—body mass index.

## Data Availability

The raw data supporting the conclusions of this article will be made available by the authors upon request.

## Supplementary Materials

The following supporting information can be downloaded at https://www.mdpi.com/article/10.3390/ijms26073061/s1. Table S1. The list of primers used for real-time qPCR reactions in the study.

## Abbreviations

ADAM10	A disintegrin and metalloproteinase domain-containing protein 10
ADAM10/17	A disintegrin and metalloproteinase domain-containing protein 10/17
AGER	advanced glycosylation end-product-specific receptor
DAMP	damage-associated molecular pattern
esRAGE	endogenous secretory RAGE
GCLC	glutamate-cysteine ligase, catalytic subunit
GSH	reduced glutathione
GSSG	glutathione disulfide (oxidised glutathione)
GSS	glutathione synthetase
HBO	hyperbaric oxygen therapy
HIFs	hypoxia-inducible factors
HSPA1A (Hsp70)	heat shock protein family A member 1A
HSPB1 (Hsp27)	heat shock protein family B, (small) member 1
HMGB1	high-mobility group box protein 1
IKK	IkappaB kinase
MAPK	mitogen-activated protein kinases
MMP-9	matrix metalloproteinase-9
NFκB	nuclear factor-κB
NOS2	nitric oxide synthase 2
PRR	pattern recognition receptors
RAGE	receptors for advanced glycation end products
RNS	reactive oxygen species
ROS	reactive nitrogen species
S100A8	S100 calcium-binding protein A8
S100A9	S100 calcium-binding protein A9
SCUBA	Self-Contained Breathing Apparatus
SIRT1	sirtuin1
SIRT3	sirtuin3
SOD	superoxide dismutase
TLR4	Toll-like receptor 4
TXN	thioredoxin
