# Supplementary material for: The Impact of Recreational Diving to a Depth of 40 m on Selected Intracellular DAMPs
Source: Int J Mol Sci. 2025 Mar 27;26(7):3061. doi: 10.3390/ijms26073061 (PMC11989067; doi:10.3390/ijms26073061)
Supplement: Supplementary file 1 [file ijms-26-03061-s001.zip › ijms-3540569-supplementary.pdf]

Table S1. The list of primers used for real-time qPCR reactions in the study.

Gene name	Gene symbol	Accession number	Primers sequences		Amplicon length
			**Forward sequence**	**Reverse sequence**	
high mobility group box 1	*HMGB1*	NM_002128.7	AACCTATATCCCTCC CAAAG	ACATCTCTCCCAGT TTCTTC	170
S100 calcium-binding protein A8	*S100A8*	NM_002964.5	GGGCAAGTCCGTGG GCATCA	TCATCCCTGTAGAC GGCATGGAA	117
S100 calcium-binding protein A9	*S100A9*	NM_002965.4	AAACACTCTGTGTGG CTCCT	TGGTCTCTATGTTGC GTTCCA	86
thioredoxin	*TXN*	NM_003329.4	ACAGCCGCTCGTCAG ACTCC	TGCAGCGTCCAAGG CTTCCT	84
heat shock protein family B, (small) member 1	*HSPB1*	NM_001540.5	ATGGCTACATCTCCC GGTGC	AGGAAACTTGGGTG GGGTCC	72
heat shock protein family A (Hsp70) member 1A	*HSPA1A*	NM_005345.6	ACTCCCGTTGTCCCA AGGCTTC	TCTGTCGGCTCCGC TCTGAGAT	147
Toll-like receptor 4	*TLR4*	NM_138557.3	TCCTGCGTGAGACCA GAAAGC	AAGGCTCCCAGGG CTAAACTC	144
receptor for advanced glycation endproducts	*AGER*	NM_00120693 2.2	CTCACGGCTGGTGTT CCCAA	GGGGTCACCATTAG CTCCGA	191
nuclear factor κB	*NFKB*	NM_00116541 2.2	CCAACAGATGGCCC ATACCTTCA	TGCTGGTCCCACAT AGTTGC	168
nitric oxide synthase 2	*NOS2*	NM_000625.4	GTTCTCAAGGCACAG GTCTC	GCAGGTCACTTATG TCACTTATC	127
glutamate-cysteine ligase, catalytic subunit	*GCLC*	NM_001498	GGAAGTGGATGTGG ACACCAGA	GCTTGTAGTCAGGA TGGTTTGCG	131
glutathione synthetase	*GSS*	NM_000178.4	CCAAGACCGAAGGC TGTTTGTG	TGTGACCTCTCCAG CAGTAGAC	123
